# Species D Human Adenovirus Type 9 Exhibits Better Virus-Spread Ability for Antitumor Efficacy among Alternative Serotypes

**DOI:** 10.1371/journal.pone.0087342

**Published:** 2014-02-04

**Authors:** Junji Uchino, David T. Curiel, Hideyo Ugai

**Affiliations:** 1 Cancer Biology Division, School of Medicine, Washington University in St. Louis, St. Louis, Missouri, United States of America; 2 Biologic Therapeutics Center, Department of Radiation Oncology, School of Medicine, Washington University in St. Louis, St. Louis, Missouri, United States of America; University of Pittsburgh School of Medicine, United States of America

## Abstract

Species C human adenovirus serotype 5 (HAdV-C5) is widely used as a vector for cancer gene therapy, because it efficiently transduces target cells. A variety of HAdV-C5 vectors have been developed and tested *in vitro* and *in vivo* for cancer gene therapy. While clinical trials with HAdV-C5 vectors resulted in effective responses in many cancer patients, administration of HAdV-C5 vectors to solid tumors showed responses in a limited area. A biological barrier in tumor mass is considered to hinder viral spread of HAdV-C5 vectors from infected cells. Therefore, efficient virus-spread from an infected tumor cell to surrounding tumor cells is required for successful cancer gene therapy. In this study, we compared HAdV-C5 to sixteen other HAdV serotypes selected from species A to G for virus-spread ability *in vitro*. HAdV-D9 showed better virus-spread ability than other serotypes, and its viral progeny were efficiently released from infected cells during viral replication. Although the HAdV-D9 fiber protein contains a binding site for coxsackie B virus and adenovirus receptor (CAR), HAdV-D9 showed expanded tropism for infection due to human CAR (hCAR)-independent attachment to target cells. HAdV-D9 infection effectively killed hCAR-negative cancer cells as well as hCAR-positive cancer cells. These results suggest that HADV-D9, with its better virus-spread ability, could have improved therapeutic efficacy in solid tumors compared to HAdV-C5.

## Introduction

Cancer is a major public health problem in the United States. Approximately 1.64 million new cancer cases and approximately 580,000 deaths were predicted to occur in the United States in 2012 [1]. Given this, some local therapies (surgery and radiation therapy) and systemic therapies (chemotherapy, hormone therapy, and immunotherapy) have greatly reduced the mortality of cancer disease [1]. However, primary tumors will often relapse and subsequently metastasize to other organs [2]. These considerations warrant the development of new treatment strategies for cancer.

Cancer gene therapy is one feasible treatment that involves introducing genetic material into human cells to eradicate cancer [3]. Virotherapy in particular represents a promising new direction for cancer gene therapy [4], [5]. This approach utilizes viruses designed to specifically kill tumor cells but not normal cells [6], [7]. Because the biology of HAdV-C5 is relatively well characterized [8], it has emerged as one of the most attractive candidate virotherapy agents, which are called oncolytic HAdVs [4]. A number of strategies to specifically kill tumor cells are based on the interacting molecular mechanisms between adenoviral biology and cellular signaling pathways associated with human cancers [5]. A variety of oncolytic HAdVs have been tested *in vitro* [9]–[14] and caused effective responses in clinical trials [15]–[23]. On the other hand, oncolytic HAdVs utilizing HAdV-C5 showed responses in limited area [24]–[26]. As a biological barrier in tumor mass hinders virus spread from infected cells [12], [27], [28], we need to develop more efficacious oncolytic HAdVs.

A number of transcriptional targeting strategies using tumor-specific promoters have been tried to improve replication specificity to limit toxicity [29]–[31]. Numerous infectivity enhancement strategies were tested for targeting cancer cells with no human coxsackie B virus and adenovirus receptor (hCAR) as a primary receptor for HAdV-C5 [32], [33]. In addition, several HAdV serotypes were tested to examine antitumor efficacy to solid tumors by *in vivo* [34]. Thus, many types of advanced oncolytic HAdVs have been reported for cancer gene therapy. We need HAdVs with better virus-spread ability to overcome a biological barrier in tumor mass. However, strategies to enhance virus-spread ability of HAdV-C5 are limited [35], [36].

A total of over 50 serotypes of HAdVs, divided into species A to G, have been identified [37]. These wild type HAdVs produce different sizes of plaques on cells [38], thus, we hypothesized that HAdVs with better virus-spread ability can be identified from alternative serotypes by comparing the size of the plaques. We examined viral spread ability *in vitro* of sixteen HAdV serotypes by plaque assay as compared with that of HAdV-C5. In this study, we report the biological and physical properties of HAdVs *in vitro*, highlighting HAdV-D9 as a candidate for cancer gene therapy in solid tumor.

## Materials and Methods

### Cell Lines

Human embryonic kidney 293 (HEK293), human lung adenocarcinoma cell line A549, human ovarian adenocarcinoma cell lines SKOV-3 and OVCAR-3, human pancreatic carcinoma cell lines BxPC-3 and MIA-PaCa-2, human breast cancer cell lines AU-565, MCF-7 and ZR-75-1, human malignant mesothelioma cell lines H2052, H2452 and MSTO-211H, human prostate cancer cell line PC-3, were obtained from the American Type Culture Collection (ATCC; Manassas, VA). Human embryonic kidney 293A was obtained from Life Technologies (Carlsbad, CA, USA). Chinese hamster ovary (CHO) cells and CHO-hCAR cells stably expressing human CAR were provided by Jeffrey M. Bergelson [39]. All cells described above except AU-565, ZR-75-1, three kinds of malignant mesothelioma cell lines, CHO, and CHO-hCAR were cultured in Dulbecco’s modified Eagle’s medium/nutrient mixture F-12 Ham, (DMEM/F12; Sigma-Aldrich, St. Louis, MO) containing 10% fetal bovine serum (FBS; Hyclone; Logan, UT), 2 mM L-glutamine, 100 U/ml penicillin, and 100 mg/ml streptomycin (Mediatech, Inc., Herndon, VA). The other cell lines were maintained in culture medium recommended by each supplier. All cells were incubated at 37°C in an atmosphere of 5% CO_2_ in air. Also, infected cells were maintained with medium containing 2% FBS, 2 mM L-glutamine, 100 U/ml penicillin, and 100 mg/ml streptomycin.

### Adenoviruses, Propagation, and Purification

Human adenoviruses (HAdVs), HAdV-C2, B3, E4, C5, D9, D10, B14, B16, D20, B21, A31, B34, B35, D37, F40, F41, and D51 were obtained from ATCC and were propagated in A549 or HEK293 cells. We purified HAdVs in ten T175-cm^2^ flasks of infected A549 or infected HEK293 cells by two rounds of cesium chloride (CsCl) gradient ultracentrifugation [40]. CsCl was removed by dialysis against phosphate-buffered saline (PBS [pH 7.4]) containing 10% glycerol. Purified HAdVs were stored at −80°C prior to experiments.

### Titration

Plaque assay was performed to determine the infectious titer (plaque forming units; PFU/ml) of purified HAdVs on 293A cells or A549 cells [41]. The particle titer (viral particles [VP]/ml) was determined by *A*_260_ absorbance of purified virus particles and by assuming that 1.1×10^12^ VP/ml has an absorbance of 1.0 at 260 nm [42]. In brief, the particle titer is calculated based on the protein amount of the purified adenoviruses observed at absorbance at 260 nm under denaturing conditions using SDS (an OD_260_ of 1.0 corresponds to 0.28 mg/ml protein in 0.1% [w/v] SDS buffer). A value of 1.77×10^8^ daltons (Da) for the molecular mass of the VP is obtained by dividing 2.3×10^7^ Da (the molecular mass of adenovirus DNA) by 0.13 because 13% of an adenoviral particle is DNA. Next, 1.54×10^8^ Da, the molecular mass of the adenoviral proteins that compose the VP, is calculated by subtracting 2.3×10^7^ Da (the molecular mass of the adenovirus DNA) from 1.77×10^8^ Da (the molecular mass of the VP). Finally, 1.1×10^12^ particles/ml/OD_260_ unit is obtained by dividing 2.8×10^−4^ g/ml protein by 1.54×10^8^ Da (the molecular mass of the adenoviral proteins) and multiplying it with Avogadro’s number (*N_A_*) (6.02×10^23^). In order to calculate the HAdV genome titer, we extracted the adenovirus genome from purified HAdV. In brief, 100 µl of purified adenovirus were mixed with sodium dodecyl sulfate (SDS, Sigma-Aldrich) and proteinase K (Sigma-Aldrich) at final concentrations of 0.1% (w/v) and 0.1 mg/ml, respectively. The mixture was incubated at 56°C for 1 hour and subsequently cooled at room temperature. Purified adenovirus genome was extracted by a standard method with phenol and chloroform [43] and dissolved with 100 µl of sterilized H_2_O. The titer of the adenovirus genome was calculated on the assumption that a solution of 50 µg/ml of purified DNA has an absorbance of 1.0 at 260 nm [44]. We then calculated the concentration of the adenoviral genome using the following formula: adenoviral genome titer at an absorbance of 1.0 at 260 nm = (5.0×10^−5^ g/ml)/molecular mass of the double stranded adenoviral genome×Avogadro’s number (*N_A_*) = 6.02×10^23^ mol^−1^ [44]. We summarized accession numbers of the HAdV genomes which were obtained from GenBank and each genome titer per 1OD at 260 nm in Table S1.

### Measurement of Plaque Sizes

We performed plaque assay as described elsewhere [41] and measured the sizes of individual single plaques by Traceable Certificate of Calibration for Digital Caliper (Control Company, Friendswood, TX, USA). In brief, a solution of purified HAdVs was subjected to 10-fold serial dilution and A549 cells or 293A cells were infected with the diluted HAdVs in a six-well plate. After 1 hour post-infection, each solution of HAdVs was removed and 2 ml of fresh DMEM/F12 that contained 2% FBS and 0.75% BD Difco agar (BD Diagnostic Systems, Sparks, MD, USA) was laid over infected cells. Subsequently we incubated infected cells and laid over 2 ml of fresh DMEM/F12 that contained 2% FBS and 0.75% agar at 4 and 8 days post-infection. We culture infected cells another six days and laid over 2 ml of fresh DMEM/F12 that contained 2% FBS, 0.75% agar, and 0.033% Neutral red (Life Technologies, Carlsbad, CA, USA). We measured sizes of 10 individual single plaques which were visualized by neutral red staining and calculated the mean of plaque sizes. In cases of HAdV-B16, F40 and F41, we were not able to measure the sizes of plaques by a caliper due to their very small sizes. Therefore, we counted the number of plaques produced with these HAdVs on cells using an inverted IX-70 microscope (Olympus Corporation, Melville, NY) equipped with a DP71 digital camera (Olympus Corporation) and software of DP Controller and DP Manager (Olympus Corporation).

### One-step Growth Curve Analysis of HAdVs

A549 cells were grown to 80% confluence in 6-well plates and the number of cells was counted to determine a multiplicity of infection (MOI). Cells were infected with HAdV at an MOI of 10 PFU/cell. Infected cells were maintained in 3 ml of medium containing 2% FBS and both infected cells and culture medium were harvested by using a cell scraper at various hours post-infection. We disrupted infected cells along with culture medium by three cycles of freeze and thaw and removed cell debris by centrifugation at 1,000×g for 5 minutes (min) at 4°C. The supernatant was used as the sample of whole HAdV contained in the fraction of both infected cells and culture medium. Additionally, we separated culture medium and infected cells by centrifugation at 1,000×g for 5 min at 4°C. The culture medium was used as a solution of HAdV which was released from infected cells. The infected cell pellet was resuspended in 2 ml of fresh medium and disrupted by three cycles of freeze and thaw. The lysates were centrifuged at 3,500×g for 10 min at 4°C and the supernatant was used as a sample of HAdV which was held in infected cells. Solutions of HAdVs prepared from each fraction were stored at −80°C prior to titration.

### Cellular Uptake of HAdV

Cells were grown to 80% confluency in 24-well plate and the number of cells was counted before viral infection. We infected cells with HAdV at an MOI of 100 genomes/cell and maintained infected cells in 1 ml of medium for 30 or 60 min. After 30 or 60 min post-infection, infected cells were harvested and centrifuged 1,000×g for 5 min at 4°C. For a blocking experiment, CHO-hCAR cells were grown to 80% confluency in 24-well plate and removed medium. We incubated CHO-hCAR cells with 1 ml of FBS-free medium containing recombinant HAdV-C5 fiber knob protein [45] at a final concentration of 0.5, 5.0 or 50 µg/ml at 4°C for 1 hour. No blocking agent was added to the control wells. After 1 hour post-incubation, we removed medium and infected cells with HAdV-C5 or HAdV-D9 at an MOI of 100 genomes/cell for 1 hour. Subsequently, we harvested infected cells and medium, centrifuged 1,000×g for 5 min at 4°C, and removed supernatant. Total cell DNA containing adenoviral genome was extracted from infected cells by a method described previously [46] and stored at −80°C prior to quantitative PCR (qPCR) analysis.

### Quantitative PCR (qPCR) Analysis

Position 1 refers to the left end of wild-type HAdV-C5 genome (GenBank accession number AC_000008.1), wild-type HAdV-D9 genome (accession number AJ854486.1). Oligonucleotides corresponding to the sense strand of the HAdV-C5 L4 region (5′-GAGCGCTCAGGAATCTTGC-3′: 25,576–25,594 nucleotide position [nt]), the antisense strand of the L4 region (5′-CGCGGTACTTAATGGGCAC-3′; 25,645–25,627 nt), the sense strand of the HAdV-D9 E3 region (5′-GTCCCATGGTGACTCTGCT-3′: 26,186–26,204 nt), the antisense strand of the E3 region (5′-CAGTGGTCCAGATGCCTCA-3′; 26,236–26,218 nt). The qPCR condition was as follows: 40 cycles of denaturation at 94°C for 15 seconds (sec) and annealing and extension at 60°C for 60 sec. Each virus genome extracted from purified HAdVs was used to generate a standard curve to calculate the adenoviral L4 or E3 DNA copy number. We calculated a value to convert one copy number of the HAdV-C5 genome to the copy number of the HAdV-D9 genome by using the standard curves of HAdV-genomes (Figure S1). As a result, one copy number of the HAdV-C5 genome is equivalent to 1.04 of the HAdV-D9. We normalized cellular uptakes of HAdVs in CHO and CHO-hCAR as a fold of genome transfer detected in HAdV-C5-infected CHO cells at 1 hour post-infection. Triplicates of analysis were performed with 50 ng of total DNA extracted from infected cells by using SYBR Select Master Mix (Life Technologies).

### Flow Cytometry

Cells were cultured in a 75-cm^2^ tissue culture flask, harvested with PBS [pH 7.4] containing 0.53 mM EDTA, and washed with wash buffer (PBS [pH 7.4] containing 0.1% bovine serum albumin [BSA]). We incubated 5.0×10^4^ cells with anti-human CAR monoclonal antibody RmcB [47] which was kindly provided from Dr. Douglas [48] for 1 hour. Following incubation, the cells were washed three times with wash buffer and incubated with Alexa Fluor 488-conjugated goat anti-mouse immunoglobulin G (Invitrogen). After two additional washing steps, the cells were analyzed using MACSQuant Analyzer (Milteryl Biotec Inc. Auburn, CA) or Becton Dickinson Fluorescence Activated Cell Sorter (FACS aria) (BD Biosciences, Franklin Lakes, NJ).

### Two-dimensional Cell Viability Assay

Cells were seeded at a density of 5.0×10^3^ cells per well in 96-well culture plates and infected with adenoviruses at MOIs of serial PFU/cell. We assessed cytopathic effect induced with HAdV infection at 6 days post-infection by using CellTiter 96 AQ_ueous_ Non-Radioactive Cell Proliferation Assay Kit (Promega, Madison, WI) in accordance with the manufacture’s instruction. We measured the absorbance of the formazan product at 490 nm and the absorbance at 630 nm as a reference by a microplate reader (PowerWave HT 340, BioTek, Winooski, VT) and eliminated the value obtained at 630 nm as a background from that obtained at 490 nm. Cell killing activity induced with the HAdV infection was represented as relative value to uninfected cells by using GraphPad Prism 6 (GraphPad Software, Inc, La Jolla, CA).

### Three-dimensional Cell Viability Assay

We measured cell killing activity of HAdVs in spheroids formed by Cultrex 3-D Spheroid Colorimetric Proliferation/Viability Assay (Trevigen, Inc. Gaithersburg, MD). In brief, cells were seeded at a density of 5.0×10^3^ cells per well in a 3-D culture qualified 96-well spheroid formation plate, centrifuged at 200×*g* for 3 minutes at room temperature in a swinging bucket rotor. We incubated cells at 37°C in an atmosphere of 5% CO_2_ in air for 72 hours for spheroid formation. We counted cell numbers by trypsinizing spheroids and infected spheroids with adenovirus at various MOIs. We assessed cytopathic effect induced with HAdV infection at 12 days post-infection in accordance with the manufacture’s instruction. We measured the absorbance of the formazan product at 560 nm and the absorbance at 630 nm as a reference by PowerWave HT 340 microplate reader (BioTek) and eliminated the value obtained at 630 nm as a background from that obtained at 560 nm. Cell killing activity induced with the HAdV infection was represented as relative value to uninfected cells by using GraphPad Prism 6 (GraphPad Software).

### Statistical Analysis

The data were expressed as mean+standard deviation (SD) or mean + standard error of the mean (SEM). Unpaired student *t*-test was used for the statistical analysis.

## Results

### Biological and Physical Properties of HAdVs

We used 16 serotypes (HAdV-C2, B3, E4, D9, D10, B14, B16, B21, D20, A31, B34, B35, D37, F40, F41, and D51) selected from species A to G in order to examine virus-spread ability as compared to HAdV-C5. We summarized cellular receptors for HAdVs which were suggested due to being experimentally examined by using some HAdVs in Table S2. We first propagated HAdVs in a human lung carcinoma cell line, A549 cells, which is a permissive cell line to propagate HAdVs. Of the tested HAdVs, 10 serotypes (HAdV-C2, B3, E4, D9, D10, A31, B34, B35, D37, and D51) as well as HAdV-C5 were propagated well in A549 cells, but 6 kinds of HAdVs (HAdV-B14, B16, B21, D20, F40, and F41) were not. In contrast, these HAdVs (HAdV-B14, B16, B21, D20, F40, and F41) were propagated well in HEK293 cells (data not shown). Both A549 and HEK293 cell lines express the tested HAdVs’ receptors [49], hCAR, human CD46 (hCD46), and αv-integrins [49]. Nevertheless, the propagation of 6 kinds of HAdVs (HAdV-B14, B16, B21, D20, F40, and F41) was restricted in A549 cells. Thus, these experimental observations suggested that 6 kinds of HAdVs propagated in HEK293 cells had host range diversity for productive infection. We purified HAdVs which were propagated in ten T175-cm^2^ flasks of A549 or HEK293 cells and measured biological and physical titers of purified HAdVs. Because 6 serotypes (HAdV-B14, B16, B21, D20, F40, and F41) were not propagated well in A549 cells, we used 293A cells to measure those infectious titers by plaque assay. We were able to count the number of plaques on A549 or 293A cells infected with most HAdVs by a caliper, but not able to count the plaques of HAdV-B16, F40, and F41 due to being very small sizes (data not shown). Therefore, we counted the numbers of plaques of these HAdVs observed by microscopy (Figures S2 and S3) and then calculated their infectious titers (Table 1). The infectious titers of most HAdVs were similar to that of HAdV-C5, but those of HAdV-B3, HAdV-B16, A31, F40, and F41 were 20- to 890-fold lower as compared to HAdV-C5 (Table 1). The genome titers of most HAdVs showed similar values to that of HAdV-C5, and the ratios of genomes to PFU in HAdVs except HAdV-B3, F40, and F41 were the range of 10 to 520 (Table 1). Moreover, the values of particle titers of most HAdVs were similar to that of HAdV-C5, but the particle titers of HAdV-B16 and F40 showed 10- to 40-fold lower values than that of HAdV-C5 (Table 1). Thus, the ratios of particles to PFU in HAdVs except HAdV-A31, B3, F40, and F41 were in the range of 10 to 360 (Table 1). The analyses of the biological and physical properties of HAdVs purified in this study were reproducible. Dr. Green *et al.* have reported that the ratios of particles to PFU of HAdV-C1 to D30 which were purified from infected KB cells were the ranges from 11∶1 to 2300∶1 [38]. Thus, we obtained similar ratios of particles to PFU in HAdVs except HAdV-B3 and D21 as compared with data reported by Dr. Green *et al.* [38]. Together, our results indicated that some HAdV serotypes have host range diversity and most HAdVs were effectively purified from A549 or 293 cells (Table 1).

**Table 1:** Summary of biological and physical titers of wild type HAdVs used in this study.

Host cell line	Species	Serotypes^a^	PFU/ml	Total PFU^b^	Genomes/ml	Genomes/PFU	VP/ml	VP/PFU
A549	A	31	1.17×10^9^±7.64×10^8^	3.97×10^9^	4.48×10^11^	384	2.26×10^11^	2,513
	B1	3	2.83×10^7^±2.02×10^7^	9.63×10^7^	1.14×10^12^	40,305	3.79×10^11^	75,800
	B2	34	7.17×10^9^±1.26×10^9^	3.50×10^10^	3.92×10^11^	55	4.09×10^11^	57
		35	6.00×10^9^±5.00×10^8^	3.00×10^10^	6.90×10^11^	115	1.90×10^11^	32
	C	2	4.17×10^9^±5.77×10^8^	1.08×10^10^	2.27×10^11^	55	1.78×10^11^	43
		5	2.53×10^10^±1.53×10^9^	1.01×10^11^	5.12×10^11^	20	1.46×10^12^	58
	D	9	2.33×10^10^±1.76×10^10^	1.87×10^11^	4.64×10^12^	199	3.52×10^12^	151
		10	4.83×10^9^±2.89×10^8^	1.11×10^10^	2.49×10^12^	514	1.73×10^12^	357
		37	2.03×10^10^±4.16×10^9^	7.12×10^10^	1.37×10^12^	67	1.31×10^12^	65
		51	9.00×10^9^±1.00×10^9^	6.75×10^10^	2.49×10^12^	277	2.38×10^12^	264
	E	4	2.60×10^10^±2.60×10^9^	1.56×10^11^	1.65×10^12^	64	1.49×10^12^	57
293	B1	16	5.00×10^8^±5.00×10^8^	3.75×10^9^	1.46×10^11^	292	3.60×10^10^	72
		21	6.00×10^10^±1.32×10^10^	3.24×10^11^	1.26×10^12^	21	7.63×10^11^	13
	B2	14	1.48×10^10^±6.33×10^9^	1.30×10^11^	3.20×10^12^	216	2.28×10^12^	154
	D	20	9.33×10^10^±3.01×10^10^	9.33×10^11^	1.17×10^12^	13	9.74×10^11^	10
	F	40	8.50×10^7^±5.63×10^7^	2.30×10^8^	1.28×10^12^	15,069	1.00×10^11^	1,278
		41	1.23×10^8^±4.04×10^7^	8.26×10^8^	7.20×10^11^	5,837	5.05×10^11^	5,504

a Numbers refer to the serotypes of HAdVs.

b Total PFU was calculated with the volume of purified HAdVs.

### HAdV-D9 and D51 have Better Virus-spread Ability than HAdV-C5

**Figure 1:**
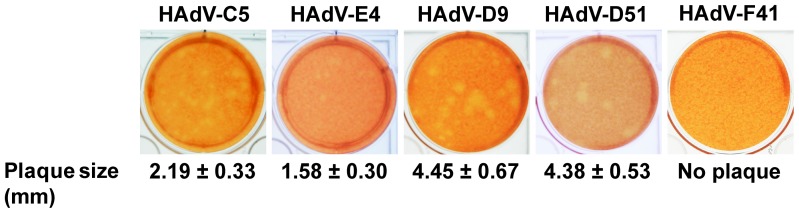
Plaque morphology of HAdVs on A549 cells. HAdVs (HAdV-C5, D9, D51, E4, and F41) were serially diluted with medium containing 2% FBS and A549 cells were infected with HAdV at the range of dilution of 5.0×10^−8^ and 5.0×10^−9^ for 1 hour. We performed plaque assay as described in the Materials and Methods section. At 14 days post-infection, we stained cells with 2 ml of medium containing 0.75% agar and 0.033% neutral red in order to visualize individual single plaques on A549 cells. Photographs of plaque morphology of HAdVs were taken with a digital camera.

In order to develop new oncolytic HAdVs with better virus-spread ability than HAdV-C5, we require HAdVs which are capable of replicating well in tumors and efficiently spreading from infected cancer cells to surrounding cancer cells. Because each serotype produces different sizes of plaques on monolayer cells [38], plaque assay is suitable for assessing virus-spread ability *in vitro* [36], [38], [50], [51]. We performed plaque assay to identify HAdVs with better virus-spread ability and summarized the plaque sizes of HAdVs which were measured by a caliper in Table 2. HAdV-D9 (mean and standard deviation of the plaque size; 4.45±0.67 mm, n = 10) and HAdV-D51 (mean and standard deviation of the plaque size; 4.38±0.53 mm, n = 10) produced larger plaques on A549 cells than other serotypes including HAdV-C5 (Figure 1 and Table 2). Also, we observed clear plaque morphologies of HAdV-D9 and D51 with larger sizes by microscopy (Figure S2). On the other hand, the sizes of the plaques of HAdV-A31, HAdV-B3, B34, B35, D10, D37, and E4 on A549 cells were smaller than those of HAdV-C5 (Figure 1 and Table 2). As expected, we did not observe the plaques of HAdV-F41 on A549 cells (Figure 1). Although we tested plaque assay of HAdV-F41 at the range of dilution between 5.0×10^−4^ to 5.0×10^−9^ on A549 cells, we did not detect any plaques of HAdV-F41 on A549 cells by microscopy (data not shown). In contrast to A549 cells, we detected the plaques of HAdV-F41 on 293A cells (Figure S3). Because HAdV-F41 lacked virus-spread ability on A549 cells, it is not suitable for an oncolytic vector to solid tumors. Thus, our data demonstrated that HAdV-D9 and D51 have better virus-spread ability than HAdV-C5. Although HAdV-D51 as well as HAdV-D9 similarly killed in most cancer cell lines, cell killing activity by HAdV-D51 was restricted in pancreatic cell lines, BxPC-3 and MIA-PaCa-2 cells as compared to HAdV-C5 and D9 (Figure S4). While the nucleotide sequences for the HAdV-D9 genome as well as the HAdV-C5 genome were available from GenBank, the HAdV-D51 genome sequence was not determined yet. Since HAdV-D9 induced cell killing at low concentrations in broad ranges of cancer cell lines, we selected HAdV-D9 rather than HAdV-D51. We further analyzed the biological properties of HAdV-D9 with better virus-spread ability as compared to HAdV-C5.

**Table 2:** Host cell lines used for viral propagation and plaque sizes of HAdVs.

Host cell line	Species	Serotypes^a^	Plaque size^b^ (mm)
A549	A	31	0.50±0.58
	B1	3	0.78±0.38
	B2	34	1.63±0.18
		35	1.45±0.52
	C	2	2.86±1.36
		5	2.19±0.33
	D	9	4.45±0.67
		10	0.73±0.59
		37	1.13±0.32
		51	4.38±0.53
	E	4	1.58±0.30
HEK293	B1	16	unmeasurable^c^
		21	4.06±0.93
	B2	14	1.35±0.28
	D	20	4.52±0.51
	F	40	unmeasurable^c^
		41	unmeasurable^c^

a Numbers refer to the serotypes of HAdVs.

b Plaque sizes of HAdVs were represented as the average of the values obtained from ten individual single plaques.

c Plaque sizes of these viruses were not able to be measured by a caliper.

### HAdV-D9 is Efficiently Released from Infected Cells

**Figure 2:**
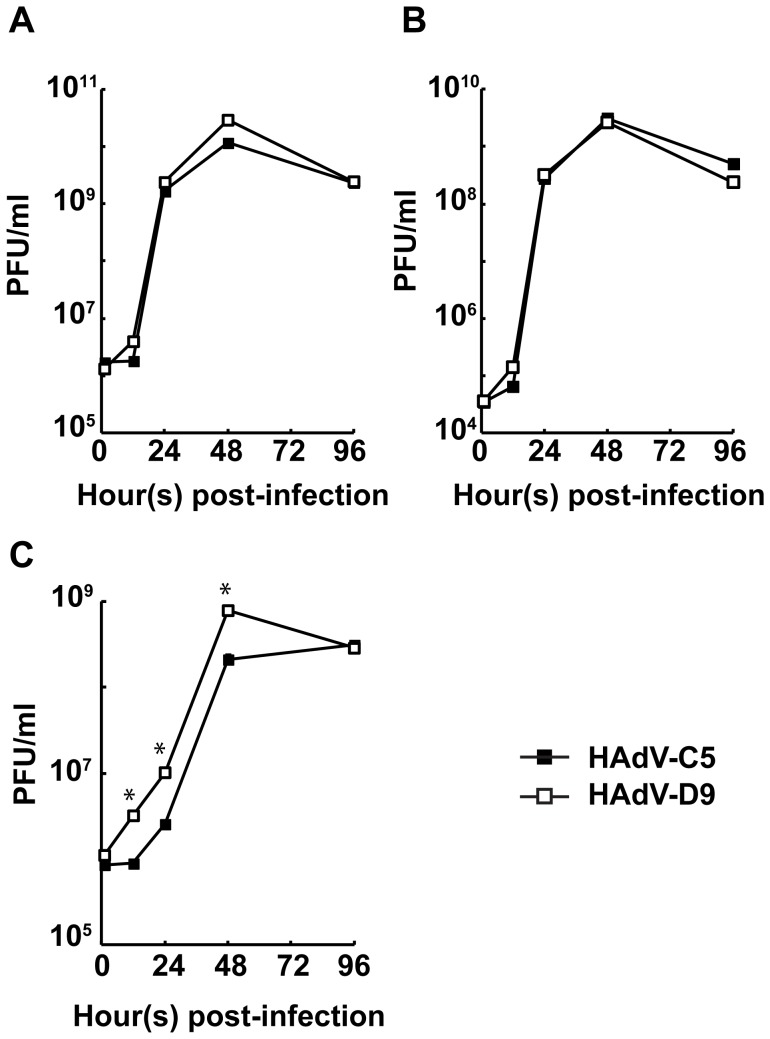
Release of HAdV-D9 from infected cells to culture medium. A549 cells were infected with HAdV-C5 or HAdV-D9 at an MOI of 10 PFU/cell and harvested at various time points. Samples to measure the infectious titer of HAdVs were prepared from infected cells harvested along with culture medium (A), a fraction extracted from infected cells without culture medium (B), and culture medium (C). Infectious titers of HAdVs contained in each fraction were measured by triplicate TCID50 assays. Data points represent mean+standard error of the mean (n = 3). Unpaired student *t*-test analysis was performed with respect to HAdV-C5 at each time point and significance is indicated by *P<0.05.

The plaque sizes of HAdV-D9 on A549 cells were larger than those of HAdV-C5 (Figure 1 and Table 2). We supposed that the release of the HAdV-D9 progeny from infected cells is better than that of HAdV-C5. Therefore, we measured infectious titers of HAdVs which were released from infected cells to culture medium at various times post-infection by plaque assay. Also, we determined infectious titers of HAdVs in infected cells and the whole of infected cells and culture medium. The replication of HAdV-D9 illustrated similar kinetics to that of HAdV-C5 in the whole of both infected cells and culture medium (Figure 2A). Also, the replication ability of HAdV-D9 in infected cells was comparable to that of HAdV-C5 (Figure 2B). As expected, HAdV-D9 was efficiently released by an earlier time point from infected cells to culture medium as compared with HAdV-C5 (Figure 2C). Moreover, the amounts of infectious HAdV-D9 which were detected in culture medium continued to be significantly higher than those of infectious HAdV-C5 during viral replication observed by 12 hours post-infection (Figure 2B and 2C). Thus, these results demonstrated that the HAdV-D9 progeny were more efficiently released from infected cells to culture medium as compared to HAdV-C5.

### HAdV-D9 Infection is Independent of hCAR

**Figure 3:**
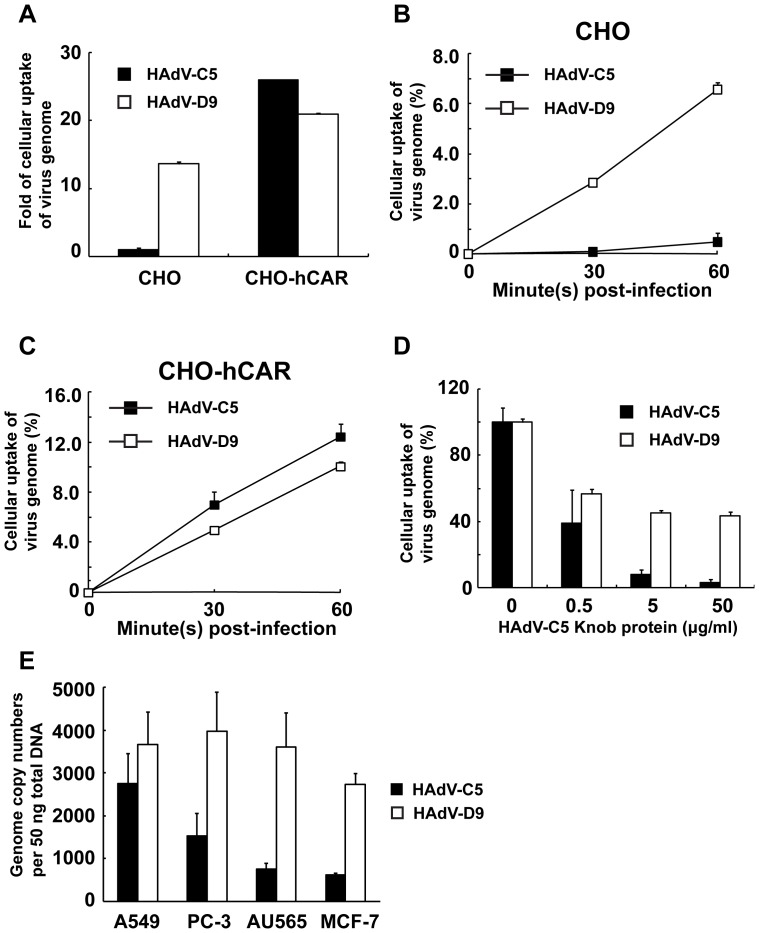
Cellular uptake of HAdV-D9 independently of human CAR. Cells (CHO and CHO-hCAR) were infected with HAdV-C5 or HAdV-D9 at an MOI of 100 genomes/cell, and total cell DNA extracted from infected cells were used for qPCR analysis. (A) Cellular uptakes of HAdVs in CHO and CHO-hCAR were represented as a fold of genome transfer in HAdV-C5-infected CHO cells at 1 hour post-infection and normalized by cellular uptake of HAdV-C5 in CHO cells; HAdV-C5 (black bars) and HAdV-D9 (white bars). (B and C) Cellular uptakes of HAdVs in a time-dependent manner in CHO (B) and CHO-hCAR (C). Total cell DNA was extracted from infected cells at 0, 30, and 60 min post-infection and used for qPCR analysis; HAdV-C5 (black squares) and HAdV-D9 (white squares). Data points represent mean+standard error of the mean (n = 3). CHO; human CAR negative CHO cells and CHO-hCAR; CHO cells stably expressing human CAR. (D) CHO-hCAR cells were treated with the HAdV-C5 fiber knob protein at a final concentration of 0, 0.5, 5.0 or 50 µg/ml at 4°C for 1 hour and then infected with HAdV at an MOI of 100 genomes/cell at 37°C for 1 hour. HAdV-C5 (black bars) and HAdV-D9 (white bars). (E) Cellular uptakes of HAdV-D9 in cancer cells which express little or no hCAR. Cells were infected with HAdV-C5 or HAdV-D9 at an MOI of 100 genomes/cell for 1 hour post-infection, and total cell DNA extracted from infected cells was analyzed by qPCR. HAdV-C5 (black bars) and HAdV-D9 (white bars). Cellular uptakes of HAdVs in cell lines were represented as (A) a fold of genome transfer, (B, C and D) a percentage of genome transfer, and (E) Genome copy numbers per 50 ng of total DNA of each HAdV. Data points represent mean+standard error of the mean (n = 3).

Species D HAdVs are considered to use several cellular receptors: hCAR, hCD46, αv-integrins, and/or sialic acids [52], [53]. Since HAdV-D9 expresses the fiber protein which contains a binding site for hCAR [54] and is capable of binding to hCAR [55], it is considered to utilize hCAR as a receptor for binding to target cells. On the other hand, HAdV-D9 is considered to possess the ability to interact with target cells independently of hCAR [53]. In addition, it has been suggested that the RGD motif of penton-base interacts directly with an integrin without the association of the fiber and hCAR [56]. Therefore, we examined the modality of HAdV-D9 infection in CHO and CHO-hCAR cells which stably express full length hCAR by quantifying the HAdV genomes. One copy number of the HAdV-C5 genome is equivalent to 1.04 copies of the HAdV-D9 genome. HAdV-D9 as well as HAdV-C5 was incorporated into CHO-hCAR cells (Figure 3A). Of note, the cellular uptake of the HAdV-D9 genome in CHO cells was approximately 15-fold higher than that of HAdV-C5 (Figure 3A). Thus, these data suggested that HAdV-D9 was more efficiently incorporated into CHO cells as compared to HAdV-C5 (Figure 3A). Also, the cellular uptake of the HAdV-D9 genome in CHO and CHO-hCAR cells was observed in a time-dependent manner (Figure 3B and 3C). On the other hand, the HAdV-C5 genome was readily detected in CHO-hCAR cells, but not in CHO cells (Figure 3B and 3C). Therefore, these results suggested that HAdV-D9 infection to target cells is independent of hCAR. We also investigated HAdV-D9 binding to CHO-hCAR cells with or without a blocking of the HAdV-C5 recombinant knob protein. While the recombinant protein blocked attachment of HAdV-C5 to CHO-hCAR cells in a dose dependent manner, it only partially inhibited that of HAdV-D9 (Figure 3D). This result was identical with an observation previously reported in an hCAR-positive cancer cell line [56]. Thus, the blocking of hCAR mediated by the HAdV-C5 recombinant knob protein did not inhibit HAdV-D9 infection. Moreover, we assessed whether HAdV-D9 targets human cancer cells expressing little or no hCAR as well as hCAR-positive A549 cells. The infection of HAdV-D9 in A549 cells was approximately 1.3-fold higher than that of HAdV-C5. In PC-3, AU-565 and MCF-7 cell lines, which express little or no hCAR [49], [57], it was approximately 2- to 4-fold higher than that of HAdV-C5 (Figure 3E). Thus, HAdV-D9 was incorporated into not only cancer cell lines which express little or no hCAR but also hCAR-positive cancer cells (Figure 3E). This data also supported the concept that the gene transfer mediated by HAdV-D9 is achieved at an hCAR-independent manner. Taken together, HAdV-D9 infection demonstrated hCAR-independent tropism in cancer cells.

### HAdV-D9 Exhibits Anticancer Effects in Variety of Cancer Cell Lines and a Spheroid Model

**Figure 4:**
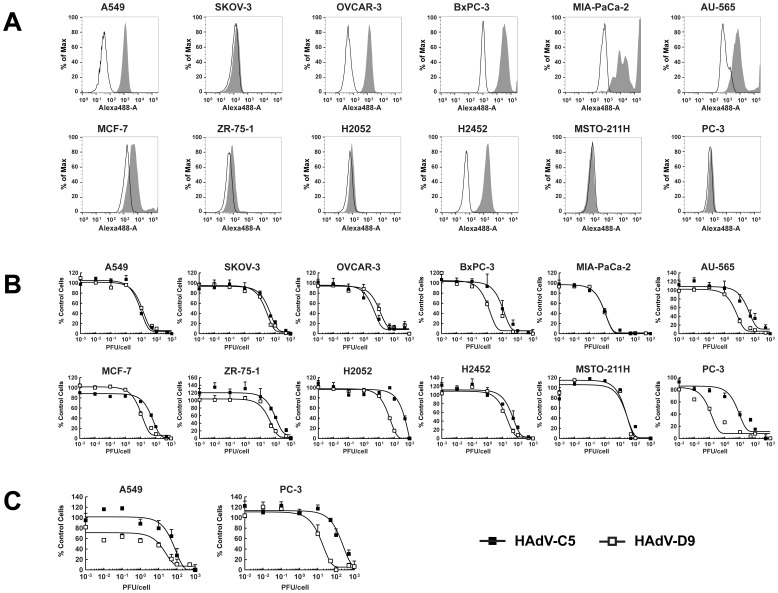
Analysis of hCAR expression and cell killing activity of HAdV-D9 in cancer cell lines. (A) Analysis of hCAR expression in various cancer cell lines by flow cytometry. Filled, gray histograms indicate stained cells; open, white histograms indicate unstained control cells. (B) Two-dimensional cell viability assay. Cancer cell lines were infected with HAdV-C5 (black squares) or HAdV-D9 (white squares) at indicated MOIs. Cell survival in each well was measured at 6 days post-infection using an MTS assay and plotted on y-axis as the percentage of the control values obtained from uninfected cells. (C) Three-dimensional cell viability assay. Spheroids were infected with HAdV-C5 (black squares) or HAdV-D9 (white squares) at indicated MOIs. Cell survival in each well was measured at 12 days post-infection using an MTT assay and plotted on y-axis as the percentage of the control values obtained from uninfected cells. Data points represent mean+standard error of the mean (n = 3).

HAdV-D9 exhibits increased cellular uptake in the absence of hCAR and demonstrated hCAR-independent tropism in cancer cells (Figure 3). To examine whether HAdV-D9 infection kills hCAR-negative cancer cells, we performed *in vitro* cell killing assay in a broad range of cancer cell lines including hCAR-positive cancer cell lines. Cell killing activity of HAdV-D9 in these cell lines was determined by measuring remaining cell viability at 6 days post-infection. We first tested hCAR expression in cancer cell lines by flow cytometry using anti-hCAR, clone RmcB [58]. A549, OVCAR-3, BxPC-3, and H2452 cells expressed hCAR at high levels (Figure 4A). While MIA-PaCa-2 and AU-565 cells expressed hCAR at middle levels, MCF-7, ZR-75-1, and H2052 cells expressed hCAR at very low levels [59] (Figure 4A). On the other hand, hCAR expression in SKOV-3, MSTO-211H, and PC-3 cells was undetectable (Figure 4A). HAdV-D9 was able to induce cell killing at smaller amounts of infectious viruses in BxPC-3, AU-565, MCF-7, ZR-75-1, H2052 and PC-3 as compared to HAdV-C5 (Figure 4B). Also, HAdV-D9 as well as HAdV-C5 similarly killed the other cancer cell lines (Figure 4B). These data demonstrated that HAdV-D9 infection effectively kills cancer cells with attenuated hCAR and as well as hCAR-positive. Moreover, we evaluated cell killing activity of HAdV-D9 in spheroids of A549 or PC-3 cells. HAdV-D9 induced cell killing at smaller amounts of infectious viruses in PC-3 spheroids as well as A549 spheroids (Figure 4C), whereas HAdV-C5 showed attenuated cell killing activity in hCAR-negative spheroids (Figure 4C). Collectively, our data demonstrated that HAdV-D9 exhibits anticancer efficacy in broad ranges of cancer cell lines and in an *in vitro* 3-D model mimicking solid tumors.

## Discussion

A variety of oncolytic HAdVs engineered using HAdV-C5 have been characterized *in vitro* and *in vivo* [9]–[11]. In particular, 12 kinds of oncolytic HAdVs tested in patients with a variety of cancers were well tolerated [12], [15]–[23]. Most patients showed stable disease defined in Response Evaluation Criteria in Solid Tumor (RECIST) [12], [15]–[23]. On the other hand, intratumoral administration to solid tumors of an oncolytic HAdV seemed to be inefficient because tumor sizes in patients were not reduced [24]. To explain the inefficiency, a biological barrier in tumor mass is considered to hinder viral spread from infected cells [12], [27], [28]. Adenovirus mutants with improved replication ability and enhanced virus-spread ability were developed by bioselection approaches [35], [36]. Also, several HAdV serotypes were tested for identifying new HAdVs with lower seroprevalence and equal or better antitumor efficacy to solid tumors by *in vitro* cell killing assay [34]. Although HAdV-C6 was suggested as a means to address to solid tumor treatment, it showed anti-cancer activity comparable to HAdV-C5 [34].

In this study, we focused on virus-spread ability of HAdVs to overcome a biological barrier in tumor mass. We tested 16 serotypes along with HAdV-C5 in order to examine virus-spread ability. Ten of 16 serotypes, as well as HAdV-C5, propagated well in A549 cells (Table 1), but the others did not propagate (data not shown). Although HAdV-B3 was propagated in A549 cells, it showed higher values in the ratios of genomes to PFU and VP to PFU (Table 1). Our data suggested that larger numbers of the HAdV-B3 particles are needed to produce one plaque as compared with HAdV-C5. Previous reports demonstrated that larger amounts of incomplete HAdV-B3 particles are produced in infected cells [60]. Also, it is difficult to separate incomplete and infectious virus particles by purification [60]. Thus, purified HAdV-B3 which we prepared in this study may contain larger numbers of incomplete viral particles. Moreover, our data showed that HAdV-B3 and B16 were purified at very low infectious titers as compared to HAdV-C5 (Table 1). HAdV-B3 and B16 had attenuated virus-spread ability evidenced by the production of small plaques (Table 2). On the other hand, species F HAdVs show host range diversity in cell culture, and it is difficult to propagate and amplify species F HAdVs [61], [62]. Also, we had difficulty propagating species F HAdVs as well as B14, B16, D20, and B21 in A549 cells. Collectively, nine serotypes (HAdV-C2, E4, D9, D10, A31, B34, B35, D37, and D51), as well as HAdV-C5, were efficiently purified at high infectious titers from infected A549 cells and seemed to merit careful investigation for virus-spread ability.

We then investigated whether HAdV serotypes have better ability with regard to virus-spread by comparing plaque morphology with HAdV-C5 and found that HAdV-D9 and D51 generated larger plaques than those of other serotypes tested (Figure 1 and Figure S2). HAdV-D9 and D51 were screened as the candidates to make oncolytic HAdVs due to abilities of efficient production and better virus spread in cancer cells. Moreover, a seroprevalence study in a Belgian population demonstrated that HAdV-D9 and D51, as well as HAdV-C6, shows low seroprevalence as compared to HAdV-C5 [63]. These HAdVs may be suitable as gene delivery vectors for treating patients with pre-existing immunity [63]–[65]. On the other hand, HAdV-D9 was isolated from stool in 1957 [66], and HAdV-D51 was isolated from a stool specimen of an AIDS patient with fever, *Pneumocystis jirovecii* pneumonia, and diarrhea in 1989 [67]. Recently, HAdV-D9 was suggested to be associated with acute gastroenteritis in Bangladesh, as it was isolated from stools of infants and children with acute gastroenteritis at a frequency of 0.7% (6/917 patients) along with HAdV-F40 (0.8% [7/917 patients]) and HAdV-D10 (0.4% [4/917 patients]) [68]. Thus, both viruses seem to be associated with diarrhea in children and AIDS-patients. However, there are no serious diseases associated with HAdV-D9, HAdV-D51 nor HAdV-C5 [69]. Although some serotypes in species D (HAdV-D8, D19, and D37) cause epidemic keratoconjunctivitis (EKC) in humans, HAdV-D9 does not [70], [71]. Thus, HAdV-D9 is considered to be distinct from EKC-associated HAdVs [72]. The pathogenic significance of AIDS-associated HAdV infections, including HAdV-D51 in AIDS patients is unclear [73]–[75]. Since the complete nucleotide sequence of the HAdV-D9 genome, but not the HAdV-D51 genome, has been available from GenBank, the HAdV-D9 genome is suitable for characterization of the biology and vector construction. In addition, HAdV-D51 attenuated cell killing activity in pancreatic cancer cell lines as compared to HAdV-D9 (Figure S4). Based on these considerations, we characterized the biology of HAdV-D9 identified as a candidate of oncolytic HAdV vectors. HAdV-D9 as well as HAdV-C5 replicated well in A549 cells and the viral progeny was efficiently released from infected cells as compared to HAdV-C5 (Figure 2). Our results demonstrated that better ability of HAdV-D9-spread was correlated with viral release from infected cells to culture medium (Figures 1 and 2).

HAdVs involved in species D utilize several cellular receptor(s) which are hCAR, hCD46, integrins, and/or sialic acids for attachment to target cells [53]. Although several serotypes involved in species D use sialic acid as a cellular receptor [76], [77], HAdV-D9 does not use it as a receptor [76]–[78]. The interaction to hCD46 of HAdV-D9 by comparing species B HAdVs was not shown [79]. Computational analysis of the HAdV-D9 genome suggested that the fiber protein contains an hCAR-binding domain [55]. While HAdV-D9 uses hCAR at low affinity for attachment as compared to that of HAdV-C5 [55], [56], it is able to attach to αv-integrins through its penton-base at fiber-independent manner [56]. Thus, HAdV-D9 is considered to utilize both hCAR and αv-integrins for attachment [56] and preferentially use αv-integrins for attachment [54]. Although HAdV-D9 binding to hCAR-positive cells was partially blocked by the HAdV-C5 recombinant knob protein as compared to HAdV-C5 (Figure 3D) as previously reported [56], our qPCR analysis of HAdV binding to CHO cell lines and cancer cell lines with low hCAR expression demonstrated that HAdV-D9 was capable of binding to target cells independently of hCAR (Figure 3). Thus, we obtained a result that HAdV-D9 uses cellular receptor(s) other than hCAR for attachment.

One of the problems using oncolytic HAdV-C5 vectors is a low infectivity to hCAR-negative cancer cells [39], [80], [81]. The hCAR expression levels tend to be low in the majority of advanced tumors [82]–[89]. In addition, the hCAR expression in tumors exhibits marked distributional heterogeneity [89]. Thus, the varied and insufficient expression of hCAR hampers uniform HAdV-C5 transduction throughout tumors, especially in advanced tumors. Therefore, we need to target not only hCAR-positive cancer cells but also hCAR-negative cancer cells. Since HAdV-D9 efficiently killed hCAR-negative cancer cell lines as compared to HAdV-C5 (Figure 4), the expanded tropism of HAdV-D9 with better virus-spread ability will be useful for treatments of solid tumors. Another of the problems is that the majority of the HAdV-C5 vectors infect the liver by coagulation factor X, which is a vitamin K-dependent coagulation factor and the major factor in hCAR-independent uptake by hepatocytes, when they are systemically delivered [90]. However, HAdV-D9 does not bind to coagulation factor X [91], giving HAdV-D9 another advantage as an oncolytic vector over HAdV-C5, in addition to the better virus spread ability and expanded tropism of HAdV-D9.

In summary, we have selected HAdV-D9 from 16 serotypes of HAdV by comparing *in vitro* virus-spread ability as well as the *in vitro* propagation property between a cancer cell line and a cell line immortalized by the HAdV-C5 E1 gene region. Also, we revealed that a broad range of cancer cell lines were infected with HAdV-D9 independently of hCAR status. HAdV-D9 infection efficiently induced cytopathic effect in 2- and 3-D culture cells. In this study, we examined HAdV-D9 biology *in vitro* for a vector development of cancer gene therapy. Construction of the HAdV-D9 vectors is under way in our laboratory in order to overcome the limitation of HAdV-C5 vector. Our experimental results provided great impact to the development of HAdV-D9-based vectors in human gene therapy as well as the elucidation of HAdV-D9 biology on the molecular mechanism of its infection.

## Supporting Information

Figure S1 **Standard curves of the HAdV-C5 and D9 genomes for qPCR.** The copy numbers of purified HAdV genomes was calculated as described in the Materials and Methods section. A 100-fold serial dilution of purified HAdV genomes ranging from 10^2^ to 10^8^ copies per reaction was used to generate both plots; HAdV-C5 (black squares) and HAdV-D9 (white squares). Each data point represents the threshold cycle (Ct) average of samples prepared in triplicate. One copy number of the HAdV-C5 genome is equivalent to 1.04 copies of the HAdV-D9 genome.
(TIF)

Figure S2 **Comparative analysis of plaque morphology of HAdVs on A549 cells.** Plaque assay was performed as described in the Materials and Methods section. Monolayers of A549 cells in a six-well plate were infected with HAdVs, overlaid with 0.75% agar in growth medium containing 2% FBS, and stained with 0.033% neutral red at 14 days post-infection. The pictures showed microscopic view of three individual plaques formed on A549 cells infected with HAdVs.
(TIF)

Figure S3 **Comparative analysis of plaque morphology of HAdVs on 293A cells.** Monolayers of 293A cells in a six-well plate were infected with HAdVs which were propagated in 293A cells. After 1 hour post-infection, infected 293A cells were overlaid with medium containing 0.75% agar and stained with 0.033% neutral red at 14 days post-infection. The pictures showed microscopic view of three individual plaques formed on 293A cells infected with HAdVs.
(TIF)

Figure S4 **Cell killing activity of HAdV-D9 and D51 in cancer cell lines.** Nine cancer cell lines were infected with HAdV-C5 (black squares), HAdV-D9 (white squares) or HAdV-D51 (black diamonds) at indicated MOIs. Cell survival in each well was measured at 6 days post-infection using MTS assay and plotted on y-axis as the percentage of the control values obtained from uninfected cells. Data points represent mean + standard error of the mean (n = 3).
(TIF)

Table S1 **Genome copy numbers of HAdVs at an absorbance of 1.0 at 260 nm.**
(DOC)

Table S2 **Classification and cellular receptors of HAdVs.**
(DOC)
