# Supplementary figures and images for: Species D Human Adenovirus Type 9 Exhibits Better Virus-Spread Ability for Antitumor Efficacy among Alternative Serotypes
Source: PLoS One. 2014 Feb 4;9(2):e87342. doi: 10.1371/journal.pone.0087342 (PMC3913592; doi:10.1371/journal.pone.0087342)

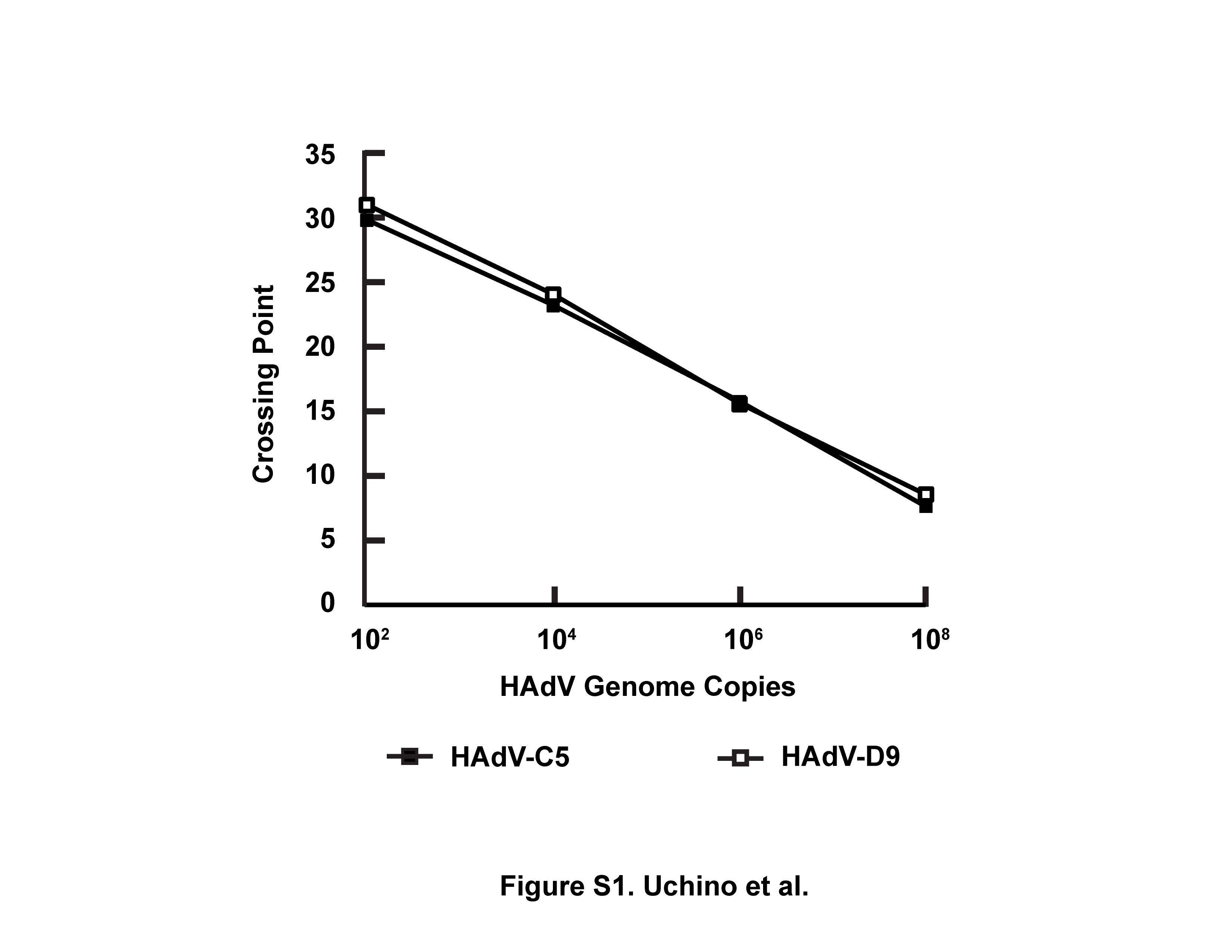

Supplement: Figure S1 — Standard curves of the HAdV-C5 and D9 genomes for qPCR. The copy numbers of purified HAdV genomes was calculated as described in the Materials and Methods section. A 100-fold serial dilution of purified HAdV genomes ranging from 102 to 108 copies per reaction was used to generate both plots; HAdV-C5 (black squares) and HAdV-D9 (white squares). Each data point represents the threshold cycle (Ct) average of samples prepared in triplicate. One copy number of the HAdV-C5 genome is equivalent to 1.04 copies of the HAdV-D9 genome. (TIF) [file pone.0087342.s001.tif]

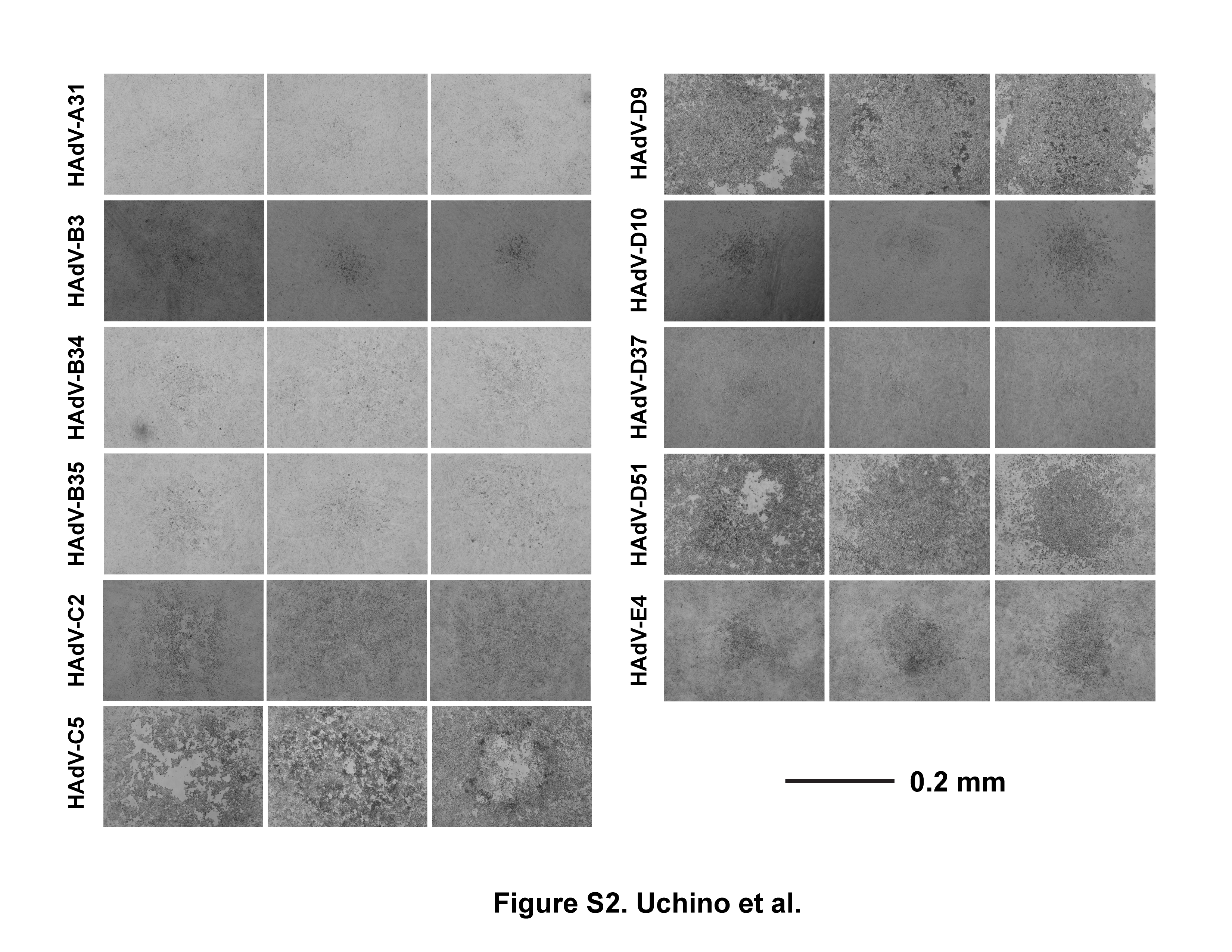

Supplement: Figure S2 — Comparative analysis of plaque morphology of HAdVs on A549 cells. Plaque assay was performed as described in the Materials and Methods section. Monolayers of A549 cells in a six-well plate were infected with HAdVs, overlaid with 0.75% agar in growth medium containing 2% FBS, and stained with 0.033% neutral red at 14 days post-infection. The pictures showed microscopic view of three individual plaques formed on A549 cells infected with HAdVs. (TIF) [file pone.0087342.s002.tif]

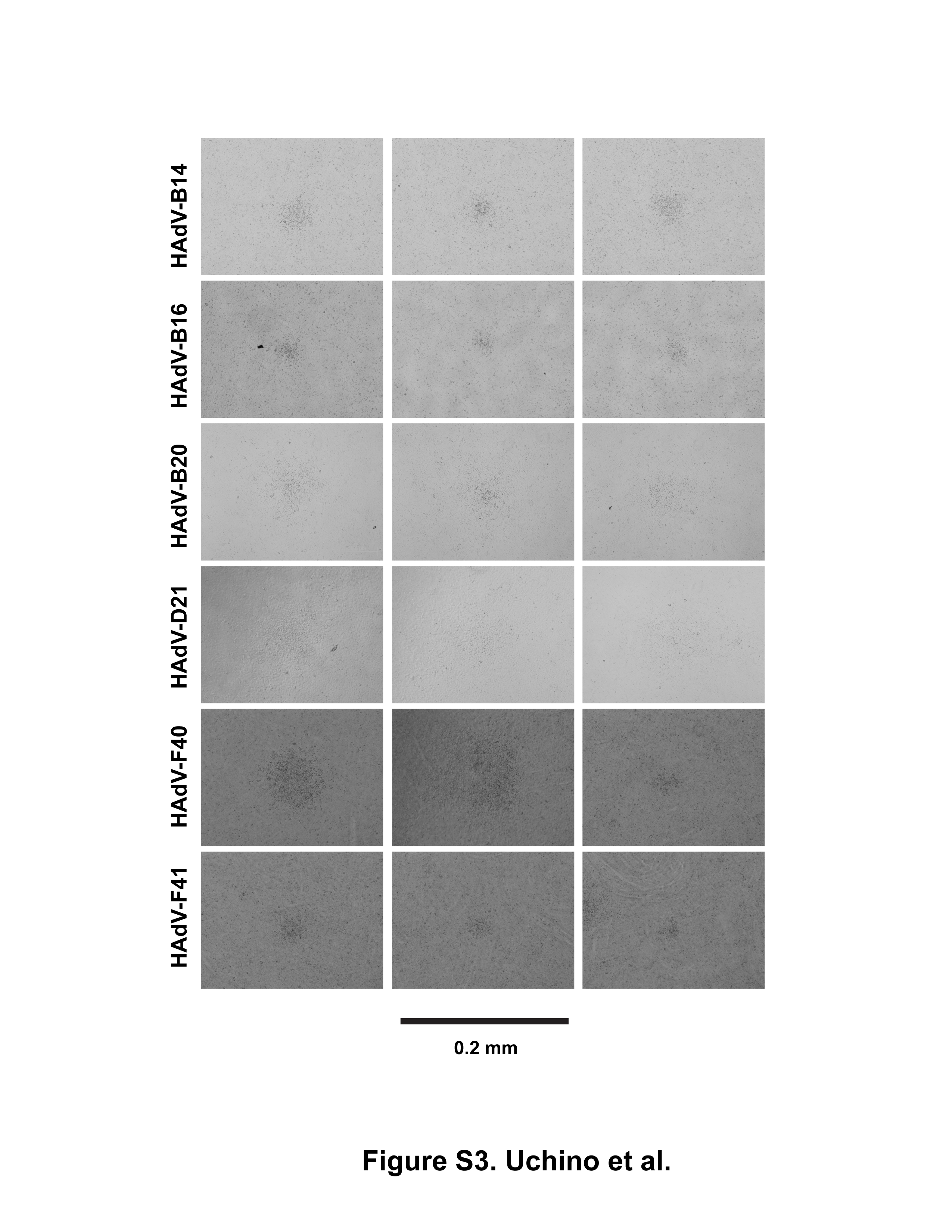

Supplement: Figure S3 — Comparative analysis of plaque morphology of HAdVs on 293A cells. Monolayers of 293A cells in a six-well plate were infected with HAdVs which were propagated in 293A cells. After 1 hour post-infection, infected 293A cells were overlaid with medium containing 0.75% agar and stained with 0.033% neutral red at 14 days post-infection. The pictures showed microscopic view of three individual plaques formed on 293A cells infected with HAdVs. (TIF) [file pone.0087342.s003.tif]

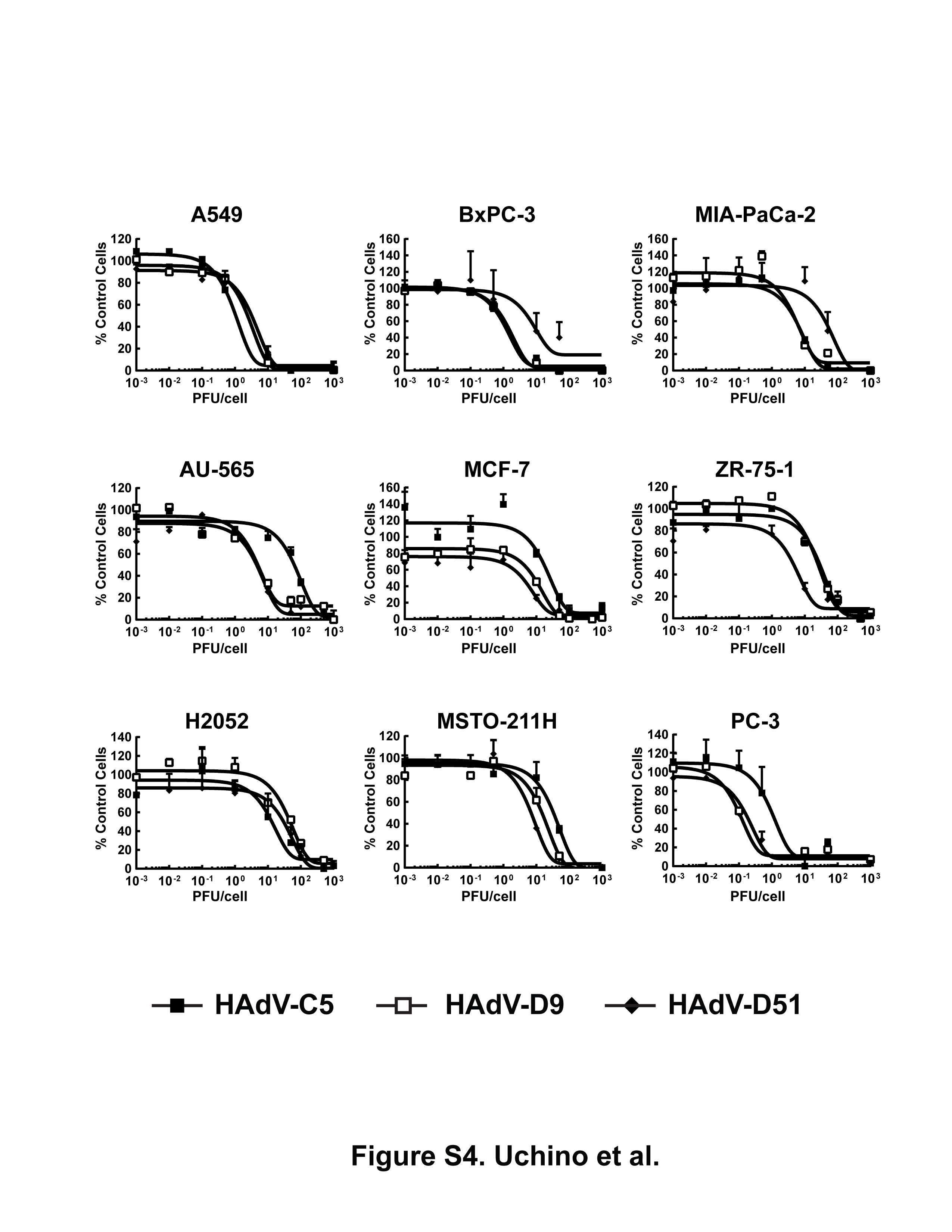

Supplement: Figure S4 — Cell killing activity of HAdV-D9 and D51 in cancer cell lines. Nine cancer cell lines were infected with HAdV-C5 (black squares), HAdV-D9 (white squares) or HAdV-D51 (black diamonds) at indicated MOIs. Cell survival in each well was measured at 6 days post-infection using MTS assay and plotted on y-axis as the percentage of the control values obtained from uninfected cells. Data points represent mean + standard error of the mean (n = 3). (TIF) [file pone.0087342.s004.tif]
