# Supplementary material for: Species D Human Adenovirus Type 9 Exhibits Better Virus-Spread Ability for Antitumor Efficacy among Alternative Serotypes
Source: PLoS One. 2014 Feb 4;9(2):e87342. doi: 10.1371/journal.pone.0087342 (PMC3913592; doi:10.1371/journal.pone.0087342)
Supplement: Table S1 — Genome copy numbers of HAdVs at an absorbance of 1.0 at 260 nm. (DOC) [file pone.0087342.s005.doc]

**Table S1. Genome copy numbers of HAdVs at an absorbance of 1.0 at 260 nm**

| Species | Serotypes^a^ | Accession number^b^ | Genome size (bp) | Genomes per 1OD at 260 nm |
| --- | --- | --- | --- | --- |
| A | 31 | AM749299.1 | 33,763 | 1.32 × 10^12^ |
| B1 | 3 | NC_011203.1 | 35,343 | 1.26 × 10^12^ |
| B2 | 34 | AY737797.1 | 34,775 | 1.28 × 10^12^ |
|  | 35 | AC_000019.1 | 34,794 | 1.28 × 10^12^ |
| C | 2 | AC_000007.1 | 35,937 | 1.24 × 10^12^ |
|  | 5 | AC_000008.1 | 35,937 | 1.24 × 10^12^ |
| D | 9 | AJ854486.1 | 35,083 | 1.27 × 10^12^ |
|  | 10 | AB695621.1 | 35,105 | 1.26 × 10^12^ |
|  | 37 | DQ900900.1 | 35,213 | 1.26 × 10^12^ |
|  | 51 | JN226765.1 | 35,114 | 1.26 × 10^12^ |
| E | 4 | AY594253.1 | 35,990 | 1.23 × 10^12^ |
| B1 | 16 | AY601636.1 | 35,522 | 1.25 × 10^12^ |
|  | 21 | AY601633.1 | 35,382 | 1.25 × 10^12^ |
| B2 | 14 | AY803294.1 | 34,764 | 1.28 × 10^12^ |
| D | 20 | JN226749.1 | 35,181 | 1.26 × 10^12^ |
| F | 40 | NC_001454.1 | 34,214 | 1.30 × 10^12^ |
|  | 41 | HM565136.3 | 34,169 | 1.30 × 10^12^ |

^a^Numbers refer to the serotypes of HAdVs

^b^Accession numbers were obtained from GenBank.
