# Supplementary material for: Species D Human Adenovirus Type 9 Exhibits Better Virus-Spread Ability for Antitumor Efficacy among Alternative Serotypes
Source: PLoS One. 2014 Feb 4;9(2):e87342. doi: 10.1371/journal.pone.0087342 (PMC3913592; doi:10.1371/journal.pone.0087342)
Supplement: Table S2 — Classification and cellular receptors of HAdVs. (DOC) [file pone.0087342.s006.doc]

Table S2. Classification and cellular receptors of HAdVs.

| Species | Serotypes^a^ | Sites of infection | Receptor (serotypes) | References^b^ |
| --- | --- | --- | --- | --- |
| A | 12, 18, 31 | Gastrointestinal tract | CAR |  |
| B1 | 3, 7, 16, 21, 50 | Lung, urinary tract | CD46 (16, 21, 50), CD80 (3), CD86 (3), Desmoglein 2 (3, 7) |  |
| B2 | 11, 14, 34, 35, 55 | Lung, urinary tract | CD46, Desmoglein 2 (11, 14) |  |
| C | 1, 2, 5, 6, 57 | Upper respiratory tract | CAR |  |
| D | 8, 9, 10, 13, 15, 17, 19, 20, 22-29, 30, 32, 33, 36, 37, 38, 39, 42-49, 51,53, 54, 56 | Eye, Gastrointestinal tract | CAR, Sialic Acid (8, 19, 37), CD46 (37) |  |
| E | 4 | Respiratory tract | CAR |  |
| F | 40, 41 | Gastrointestinal tract | CAR |  |
| G | 52 | Gastrointestinal tract | Unknown | Not reported |

^a^Numbers refer to serotypes of HAdVs.

^b^References for the HAdV receptors.

**References**

1. Roelvink PW, Lizonova A, Lee JG, Li Y, Bergelson JM, et al. (1998) The coxsackievirus-adenovirus receptor protein can function as a cellular attachment protein for adenovirus serotypes from subgroups A, C, D, E, and F. J Virol 72: 7909-7915.

2. Bewley MC, Springer K, Zhang YB, Freimuth P, Flanagan JM (1999) Structural analysis of the mechanism of adenovirus binding to its human cellular receptor, CAR. Science 286: 1579-1583.

3. Gaggar A, Shayakhmetov DM, Lieber A (2003) CD46 is a cellular receptor for group B adenoviruses. Nat Med 9: 1408-1412.

4. Marttila M, Persson D, Gustafsson D, Liszewski MK, Atkinson JP, et al. (2005) CD46 is a cellular receptor for all species B adenoviruses except types 3 and 7. J Virol 79: 14429-14436.

5. Short JJ, Vasu C, Holterman MJ, Curiel DT, Pereboev A (2006) Members of adenovirus species B utilize CD80 and CD86 as cellular attachment receptors. Virus Res 122: 144-153.

6. Wang H, Li ZY, Liu Y, Persson J, Beyer I, et al. (2011) Desmoglein 2 is a receptor for adenovirus serotypes 3, 7, 11 and 14. Nat Med 17: 96-104.

7. Segerman A, Atkinson JP, Marttila M, Dennerquist V, Wadell G, et al. (2003) Adenovirus type 11 uses CD46 as a cellular receptor. J Virol 77: 9183-9191.

8. Bergelson JM, Cunningham JA, Droguett G, Kurt-Jones EA, Krithivas A, et al. (1997) Isolation of a common receptor for Coxsackie B viruses and adenoviruses 2 and 5. Science 275: 1320-1323.

9. Wu E, Trauger SA, Pache L, Mullen TM, von Seggern DJ, et al. (2004) Membrane cofactor protein is a receptor for adenoviruses associated with epidemic keratoconjunctivitis. J Virol 78: 3897-3905.

10. Arnberg N, Edlund K, Kidd AH, Wadell G (2000) Adenovirus type 37 uses sialic acid as a cellular receptor. J Virol 74: 42-48.
